# Comparative Assesment of Fracture Resistance in Endodontically-Treated Maxillary Premolars With Different Restoration Techniques: Direct Resin Composite and Endocrown

**DOI:** 10.1155/ijod/6538214

**Published:** 2025-08-06

**Authors:** Hila Hajizadeh, Sara Majidinia, Babak Yazdani, Pegah Sadeghnezhad

**Affiliations:** ^1^Department of Restorative Dentistry, Mashhad Dental School and Dental Research Center, Mashhad University of Medical Sciences, Mashhad, Iran; ^2^Dental Materials Research Center, Mashhad Dental School, Mashhad University of Medical Sciences, Mashhad, Iran; ^3^Department of Restorative Dentistry, School of Dentistry, Mashhad University of Medical Sciences, Mashhad, Iran; ^4^Department of Dentistry, Mashhad University of Medical Science, Mashhad, Iran

**Keywords:** direct restoration, endocrown, fracture strength, resin composite

## Abstract

**Purpose:** Evaluate fracture strength in endodontically-treated maxillary premolar teeth, restored using direct resin composite or endocrowns, considering various cavity dimensions.

**Method and Materials:** Forty extracted human maxillary premolar teeth were subjected to disto-occlusal and access cavity preparation, and subsequently divided based on the cavity preparation wall thickness into 2 mm and 3 mm groups. Further, teeth were subdivided according to restoration type into: (1) direct composite, no cusp coverage; (2) direct composite, palatal cusp coverage; (3) direct composite, full-cusp coverage; and (4) lithium disilicate endocrown, full-cusp coverage (*n* = 5). Statistical analyses included the Shapiro–Wilk test, two-way ANOVA, and Tukey's post hoc test, with a significance level set at 0.05.

**Results:** Notable differences in fracture strength were observed between groups. The lowest and highest values were associated with the direct resin composite group (without cusp coverage) and the endocrown group (with full-cusp coverage), respectively. Wall thickness (2 versus 3 mm) did not significantly impact results.

**Conclusion:** In endodontically-treated maxillary premolar teeth, direct restoration with at least palatal cusp coverage or full-cusp coverage could be as successful as endocrown restoration.

## 1. Introduction

In developed countries, advancements in oral hygiene and consistent dental care have elevated tooth and dentition durability, attributed to strategic planning for oral/dental disease prevention and execution of preventive dental treatments, such as root canal therapy (RCT). Past research validates that RCTs are approximately 90% successful when administered by specialists adhering to academic guidelines [1].

In order to prevent coronal leakage and improve tooth function, it is necessary to perform an appropriate restoration after RCT [2]. Both direct and indirect methods can be used to restore endodontically treated teeth (ETT). A study by Lynch et al. [3] has shown that 91.7% of teeth restored with indirect methods, 86.5% of teeth restored with amalgam and 83% of teeth restored with resin composite were survived in an average follow-up period of 38 months. However, some studies showed the survival rate and preservation of teeth restored with the indirect method were superior than the direct methods [4–6].

Full coverage crown is one of the recommended final treatments in posterior teeth. In these cases, posts and cores are usually indicated. Endocrowns are an alternative method to these restoration. Endocrowns bond to the dentin of the pulp chamber and are often used for posterior teeth [7].

As a consequence of development of dentin bonding agents, conventional retention forms for adhesive restorations are no longer mandatory. Thus, adhesive restorations like endocrowns have the advantage of minimally invasive preparation and conservation of tooth structure [8]. However, endocrowns also have the benefit of using tooth pulp chamber walls for macromechanical retention [9]. Comparing to conventional crowns, endocrowns are easier to apply and need less preparation time [8]. A recent meta-analysis showed similar success rate between endocrowns performed in premolar and molar teeth [10].

Considering tapered roots and thin root walls of maxillary premolars, the possibility of root perforation and fracture increases during post placement. As prevention, in cases with short or curved roots, alternative treatments should be used. Among alternative treatments, direct resin composite or endocrown could be considered. As few studies have investigated the fracture strength and success rate of ETT restored with resin composite or endocrown, in this study, we aimed to compare the fracture strength of endodontically-treated maxillary premolars restored with resin composite or endocrown.

## 2. Methods and Materials

The protocol for the present laboratory study was approved by the Institutional Ethics Committee of the Faculty of Dentistry at Mashhad University of Medical Sciences with the code IR.MUMS.DENTISTRY.REC.1399.107.

Based on Rafael Francisco Lia MONDELLI study [11], 40 human maxillary premolar teeth, extracted for orthodontic reasons, were chosen and underwent disto-occlusal and standard access cavity preparations using a cylindrical diamond bur. Working lengths were determined using a #10 *K*-file and apex locator. Cleaning and shaping were carried out using the step-back technique with hand K-files up to size #35 at the apex. Irrigation was done with 5.25% NaOCl throughout instrumentation. After drying with paper points, canals were obturated using lateral compaction of gutta-percha and a resin-based sealer.

Post-RCT, they were categorized based on remaining wall thickness (2 or 3 mm), and according to restoration type into: (1) direct composite without cusp coverage; (2) direct composite with palatal cusp coverage; (3) direct composite with full-cusp coverage; and (4) lithium disilicate endocrown with full-cusp coverage.

### 2.1. Group 1 (*n* = 10)

**Figure 1:**
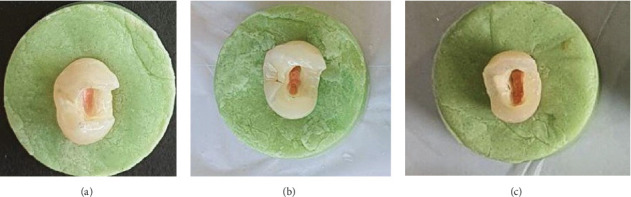
From left to right: (a) DO preparation, (b) palatal cusp coverage preparation, (c) full cusp coverage preparation (all of them had 3 mm of remaining wall thickness).

The distal proximal box was prepared up to 1 mm from the CEJ and extended towards the pulp chamber until the endodontic access was completed. Half of the samples had 2 mm of remaining wall thickness (group 1a), while the other half had 3 mm of remaining wall thickness (group 1b) (Figure 1a).

### 2.2. Group 2 (*n* = 10)

The direct composite restoration with palatal cusp coverage, in addition to group 1 preparation, the palatal cusp was reduced by 2 mm up to the mesio-palatal line angle. Half of the samples had 2 mm of remaining wall thickness (group 2a), while the other half had 3 mm of remaining wall thickness (group 2b) (Figure 1b)

### 2.3. Group 3 (*n* = 10)

The direct composite restoration group with full cuspal coverage: in addition to group 1 preparation, the entire occlusal surface was reduced by 2 mm. Half of the samples had 2 mm of remaining wall thickness (group 3a), while the other half had 3 mm of remaining wall thickness (group 3b) In both groups, cusp reduction followed anatomical guidelines, including the triangular ridge inclination, and all external line angles were beveled. (Figure 1c)

After the completion of the preparation in all these three groups of direct composite restorations, the enamel was etched for 30 s, and dentin for 15 s (Ultra-etch, Ultradent; USA). After rinsing for 10 s, they were dried with a cotton pellet to preserve the wet condition of the dentin. Subsequently, the dentin bonding agent (Ambar, FGM, Brazil) was applied in two layers with an active rubbing motion inside the cavity. Each layer was thinned with a gentle air stream for 10 s and then light cured. The curing light emitted a radiant emittance of 1000 mW/cm^2^, which is sufficient to initiate optimal polymerization of the resin composite. The curing light tip was positioned at a distance of approximately 1 mm from the resin surface to optimize light intensity while preventing direct contact that could disrupt the layer.

Each increment was irradiated for 20 s to ensure adequate depth of cure and complete cross-linking of the polymer matrix within the layer (iLED plus, Woodpecker; China).

The tofflemire matrix band with retainer was secured around the tooth, and composite resin (SDI; Australia) with an A2-dentin shade was placed in the cavity in 2 mm oblique layers, except for the first layer, which was 1 mm. Each layer was light-cured for 40 s.

The final shape was achieved using a football-shaped fine diamond bur (Jota; Switzerland). Margins and surfaces were then polished using EVE Diapol Twist spirals (EVE; Germany), which are silicone-based, diamond-impregnated polishing instruments specifically designed for polishing ceramic restorations. These instruments provide effective smoothing and high-gloss finishing, which is essential to minimize surface flaws and marginal microcracks that could compromise the long-term performance of the restoration (Figure 1).

### 2.4. Group 4 (*n* = 10)—Endocrown Group

In the endocrown group, the occlusal surface was anatomically reduced by 2 mm, and external line angles were beveled. The pulp chamber was slightly divergent through the occlusal surface. Half of the samples had 2 mm of remaining wall thickness (group 4a), while the other half had 3 mm of remaining wall thickness (group 4b).

An impression was created using the one-step putty wash method with Speedex impression material (Coltene; Switzerland). An Emax lithium disilicate endocrown (Ivoclar Vivadent; Germany) was fabricated using the heat press method with A2 low translucent (LT) ingots. After the tooth preparation, the inner surface of the endocrown was etched with 9% hydrofluoric acid (HF) (Porcelain etch, Ultradent; USA) for 45 s, following the manufacturer's instructions. It was then rinsed for 10 s. Subsequently, a layer of silane (Ultradent; USA) was applied and allowed to air dry for 1 min.

Selective enamel etching technique was employed to etch the peripheral enamel of the teeth with 35% phosphoric acid (Ultra-etch, Ultradent; USA) for 15 s.

According to the manufacturer's instructions, one drop from each of two bottles of Panavia F2.0 ED Primer was mixed together and applied to the tooth using a brush. An equal amount of A and B tubes of Panavia F2.0 cement was mixed and applied to the inner surface of the endocrown.

The endocrown was then seated on the tooth, following Turkistani et al. study [12] To ensure uniform pressure during cementation, a constant load of 5 kg (50 N) was applied to obtain standardized cement thickness. Excess cement was removed with a brush prior to light-curing. Polymerization was performed for 40 s from each direction using a curing unit with a radiant emittance of 1000 mW/cm^2^. The curing tip was positioned approximately 1–2 mm from the resin surface to maximize light intensity while avoiding direct contact.

Then remained excess cement was removed with a fine football shaped diamond bur and polished with twisted spirals.

Then specimens went under 5000 thermocycling cycles of 5–55° and UTM device (santam, Iran) was used at a speed of 0.5 mm/min with a chisel-shaped indenter with a force application angle of perpendicular to the buccal triangular ridge of palatal cusp until tooth/restoration fracture. Then the amount of fracture force was recorded in Newton (N).

A stereomicroscope with 3.5× magnification was used to evaluate the type of fracture. According to Zicari et al.'s [13] study, the type of fracture was divided into two categories: above CEJ and below CEJc.

## 3. Statistical Analysis

The Shapiro–Wilk test was used to assess the normality of data distribution. To compare fracture strengths, a two-way analysis of variance was conducted, with Tukey's post hoc test applied to make comparisons between two groups. A significance level of 0.05 was considered.

## 4. Results

According to the results of the Shapiro–Wilk test, it was found that all groups exhibited normal distribution, except for the endocrown group with full cuspal coverage in the remaining wall thickness of 3 mm.

Table 1 displays the mean, standard deviation, minimum, and maximum values of fracture strength within the groups. The results of the two-way analysis of variance test for the fracture strength variable showed that the interaction between the two factors, namely the restoration method and the remaining wall thickness, was not significant (*p*=0.692 for each). Additionally, there was no significant difference in fracture strength between the remaining wall thicknesses of 2 and 3 mm (*p*=0.506). However, there was a significant difference in fracture strength between the various restoration methods (*p*=0.011) (Table 1).

The mean fracture strength among the group of direct resin composite restoration without cusp coverage was significantly lower than the endocrown groups with full cuspal coverage and direct composite restoration with full cuspal coverage. There was no significant difference between the other groups (Table 2).

Only two specimens in the direct resin composite restoration group with a remaining thickness of 2 mm and no coverage and two specimens in the direct resin composite restoration group with a remaining thickness of 3 mm and no coverage showed fracture above the CEJ. The rest of the specimens had fractures below the CEJ.

## 5. Discussion

The study observed that groups restored with full cuspal coverage, encompassing both endocrown and direct composite restorations, demonstrated higher mean fracture strengths compared to the group that received direct resin composite restoration without cusp coverage. Apart from the noted distinction above, there were no significant differences in fracture strength among the other groups studied. This implies a level of equivalence in the performance of cusp coverage restorative approaches.

In the present study, EMAX endo crown (glass ceramic reinforced with lithium disilicate) was used, which has high stiffness and low elasticity. However, in a study by Gresnigt et al. [14] it was indicated that there was no significant difference between lithium disilicate or nanoceramic resin endocrowns.

In a study conducted by Turkistani et al. [12] on different thicknesses of lithium disilicate endocrowns placed on mandibular molars, it was concluded that the longer occlusogingival height of lithium disilicate endocrowns, the less resistance to fracture, but it has no effect on the type of failure. they also mention in their results that due to the amount of forces that endocrown can withstand, it is suggested to use it instead of traditional restorations (post and core and crown). In this study, all of the teeth had the similar crown occlusogingival height, so the study is not affected in this case.

In Gaintantzopoulou's study, intra-root expansion for endo-crown preparation has a negative effect on the marginal and internal fit of the endocrown. But using the maximum depth of the pulp chamber increases the contact surface between tooth and endocrown and reduces the risk of endocrown failure. In this study, root expansion was not used, and the cavity of the pulp chamber was prepared without undercut [15].

Another in vitro study that was conducted on molar teeth showed that increasing the depth of pulp chamber preparation extremely increases fracture resistance [15]. In another study conducted by Veneziani, it was found that in teeth with margins close to the CEJ and there is no possibility of achieving ferrule, creating a concave bevel on the peripheral enamel increases the surface of the enamel and improves the biomechanical behavior of the endocrown [16].

In Mannocci et al. [17] study, they concluded that endodontically treated premolars with loss of two or three tooth surfaces could be successfully restored with fiber post and direct resin composite without cusp coverage as final restorations. At 3-years follow-up, premolars restored with resin composite had a survival rate of more than 90%, similar to those restored with full-coverage crowns [18].

In the present study, it was found that the thickness of the remaining wall has no effect on the fracture strength of premolars. In this study, remaining wall thicknesses of 2 and 3 mm were used, the reason for not using 1 mm remaining wall thickness is the absence of dentin support, which may negatively distort the results.

Corrêa et al. [19] investigated the effect of wall thickness on the fracture strength of root-treated bovine teeth. The remaining thickness was more and less than 1 mm. Results showed that regardless of the type of restoration, tooth fracture was significantly higher in the group which remaining wall thickness was below 1 mm [19]. In Arunpraditkul et al. [20] study about the effect of the number of remaining walls of premolars on the fracture strength of teeth, they concluded that teeth with four remaining walls had significantly higher fracture resistance than teeth with three walls. However, the location of the missing wall (buccal, lingual, mesial, or distal) did not affect the fracture resistance of root-treated teeth. In another study, Pereira et al. [21] investigated the effect of remaining coronal structure on tooth fracture strength. The teeth were treated with post and resin crowns. The results showed that the remaining wall thickness did not affect the tooth fracture strength [21]. In general, it can be said that a thickness of 1 mm or less is not reliable, and there is no difference in thicker thicknesses.

According to the results of this study, the average fracture strength in the group of direct composite restoration without cusp coverage was significantly lower than the endocrown groups with full cuspal coverage and direct resin composite restoration with full cuspal coverage. Although, the amounts of endocrown fracture strength was the highest, it was not significantly different from other groups with at least palatal cusp coverage. Although, beveling is commonly discouraged in preparations for glass-based ceramics due to concerns about decreasing fracture resistance [22], in the present study, the external line angles were only minimally beveled. This beveling was not intended to create a beveled margin finish line but to round off sharp angles and reduce stress concentrations at the occlusal interface. Sharp internal or external angles can act as stress concentrators, which may negatively affect both the seating of the restoration and the distribution of occlusal forces [23]. Several authors have suggested that smoothing line angles can improve the adaptation of restorations and reduce the risk of chipping or microfracture initiation [24]. Therefore, our beveling was limited and conservative, aiming to optimize stress distribution without compromising the strength of the ceramic material. Also, it should be noted that although, the difference between the groups of composite restoration with no cusp coverage and only palatal cusp coverage wasn't significant, it was close to significance level (*p*-value = 0.054). This indicates the significance of cusp coverage in recovering the reduced fracture strength of ETT.

According to Bindl et al.[25] study, which was conducted with the aim of investigating the success rate of endocrown treatment and comparing it with conventional crowns, endocrowns can be as successful as conventional crowns in restoring root-treated teeth.

A comprehensive meta-analysis by Sedrez-Porto et al. [26] evaluated clinical (survival) and in vitro (fracture strength) studies of endocrown restorations compared to conventional treatments including intraradicular posts, direct composite resin and inlay/onlay. In general, the fracture strengths of endocrowns were significantly higher than other restorations, however, in subgroup of posterior teeth, it was found that there is no significant difference between restoration method, which might be related to low number of studies and high heterogeneity among them [26].

In the meta-analysis results of four studies by Botto et al. [27], Belleflamme et al. [28], Otto and Mormann [29], and Bindl et al. [25], there were no statistically significant difference in the rate of endocrown failure between molars and premolars. A key finding of this review was that, despite previous evidence, endocrowns on premolars may be as reliable as endocrowns on molars.

Restoration fracture was another cause of failure. A number of studies reported bulk fractures, five in molars and two in premolars. Most studies used ceramic as the material of choice for endocrowns. Ceramic materials have the advantage of stiffness and minimal elasticity, which can lead to fracture [10]. However in the present study, no bulk fracture in restoration was observed which could be due to the enough minimum thickness of 2 mm in cusp reduction and well beveled external line angles.

As an in vitro study, this study had limitations. Teeth undergo dynamic loads in oral cavity which is better to use dynamic loading instead of static loads in future studies. Also, clinical studies are needed to compare clinical performance of endocrowns versus direct composite restorations. In addition further studies, should use different ceramic materials for fabricating endocrowns.

## 6. Conclusion

The remaining wall thickness of 2 or 3 mm has no effect on the fracture strength of the teeth. Only the full cuspal coverage groups with direct restoration or endocrown led to significantly higher fracture strength than direct restoration group without cusp coverage. There was no significant difference between the other groups. According to the results of the current study, in maxillary endodontically treated premolar teeth, direct restoration with minimum coverage of the palatal cusp or full coverage could obtain similar fracture strength as endocrown.

## Figures and Tables

**Table 1:** Mean, standard deviation, minimum and maximum value of fracture strength within groups (MPa)

Group	Number	Mean	Standard deviation	Lowest	Highest
Direct composite restoration without cusp coverage	10	326.80	114.53	138.00	484.00
Direct composite restoration with palatal cusp coverage	10	476.40	137.76	209.00	681.00
Direct composite restoration with full cusp coverage	10	496.50	97.88	332.00	649.00
Endocrown with full cuspal coverage	10	594.10	252.35	328.00	1021.00

**Table 2:** Comparison of groups two by two in terms of meanfracture strength using Tukey's post hoc test.

Group	Group in comparison	*p*-Value
Direct composite restoration without cusp coverage	Direct resin composite restoration with palatal cusp coverage	0.054
	Direct composite restoration with full cusp coverage	0.030*⁣*^*∗*^
	Endocrown with full cusp coverage	0.001*⁣*^*∗*^

Direct composite restoration with palatal cusp coverage	Direct composite restoration with full cuspal coverage	0.790
	Endocrown with full cuspal coverage	0.125

Direct composite restoration with full cuspal coverage	Endocrown with full cuspal coverage	0.201

*⁣* ^*∗*^It is significant at the 5% level.

## Data Availability

The datasets used and/or analyzed during the current study are available from the corresponding author upon reasonable request.
